# Supramolecular Self-Assembled Nanostructures for Cancer Immunotherapy

**DOI:** 10.3389/fchem.2020.00380

**Published:** 2020-05-25

**Authors:** Zichao Huang, Wantong Song, Xuesi Chen

**Affiliations:** ^1^Key Laboratory of Polymer Ecomaterials, Changchun Institute of Applied Chemistry, Chinese Academy of Sciences, Changchun, China; ^2^School of Applied Chemistry and Engineering, University of Science and Technology of China, Hefei, China; ^3^Jilin Biomedical Polymers Engineering Laboratory, Changchun, China

**Keywords:** supramolecular, cancer immunotherapy, nanostructure, self-assembly, modularization

## Abstract

Functional materials and nanostructures have been widely used for enhancing the therapeutic potency and safety of current cancer immunotherapy. While profound nanostructures have been developed to participate in the development of cancer immunotherapy, the construction of intricate nanostructures with easy fabrication and functionalization properties to satisfy the diversified requirements in cancer immunotherapy are highly required. Hierarchical self-assembly using supramolecular interactions to manufacture organized architectures at multiple length scales represents an interesting and promising avenue for sophisticated nanostructure construction. In this mini-review, we will outline the recent progress made in the development of supramolecular self-assembled nanostructures for cancer immunotherapy, with special focus on the supramolecular interactions including supramolecular peptide assembly, supramolecular DNA assembly, lipid hydrophobic assembly, host-guest assembly, and biomolecular recognition assembly.

## Introduction

Cancer immunotherapy is re-defining the field of cancer therapy by activating the immune system to fight cancer. Since the approval of ipilimumab in 2011, cancer immunotherapy is playing an increasingly important role in cancer therapy (Mellman et al., 2011). Nowadays, cancer immunotherapy can be divided into three major classes by the intervention into the cancer-immunity circle: cancer vaccines, adoptive-cell transfer (ACT) therapy, and tumor immune microenvironment (TIME) modulation (Chen and Mellman, 2013; Rosenberg and Restifo, 2015; Van Der Burg et al., 2016; Zou et al., 2016). Leading products like immune checkpoint inhibitors [antibodies against programmed death 1 or its ligand (PD-1/PD-L1)] or chimeric antigen receptor T cell (CAR-T) therapy have entered the market and achieved great success in many cancer types (Hoos, 2016; June and Sadelain, 2018). However, there are still problems for current immunotherapy. One key problem is the low response rate—the overall response rate of immune checkpoint therapy in the majority of cancer types is lower than 20% (Yarchoan et al., 2017; Sanmamed and Chen, 2018), while CAR-T therapy is only effective in blood cancer (Martinez and Moon, 2019). Moreover, immune-related adverse effects (irAEs) have always been observed in clinic in association with immunotherapy, which is becoming a more and more serious problem (Hamamoto et al., 2018; Postow et al., 2018).

Nanotechnology has been widely used to enhance the therapeutic potency and safety of current cancer immunotherapy (Goldberg, 2015; Dacoba et al., 2017; Jiang et al., 2017; Song et al., 2020). Due to their unique size and surface effect, nanostructures can not only serve as carriers of small molecular drugs or proteins for targeted delivery and controlled release, but also be built with multifunctional modules to regulate the immune microenvironment in multiple dimensions (Song et al., 2017; Wang et al., 2017b; Yuan et al., 2017). In cancer vaccines, nanomaterials encapsulating tumor antigens and adjuvants can delay them from perfusion and degradation, and promote antigen presentation efficiency by an intrinsic uptake by antigen-presenting cells (APCs) and enhancing subsequent cross-presentation (Bachmann and Jennings, 2010; Fan and Moon, 2015; Irvine et al., 2015). In cell-based therapy, backpacked nanogels could promote the response of CAR-T cells in a solid tumor while *ex vivo*-generated artificial nanostructured APCs could stimulate the proliferation of tumor antigen-specific T cells *in vivo* (Wang et al., 2017a; Tang et al., 2018). In TIME modulation, the size and shape design of the nanostructures can alter the metabolic behaviors of the loaded cargos *in vivo*, which can enhance the treatment efficiency and reduce non-specific side effects (Milling et al., 2017; Ma et al., 2020a,b). As multimodular constructions, nanomaterials with multiple functional modules like antibody fragments for targeting, environmental responsive linkers for intelligent release, and secondary treatment molecules for combined therapy can deliver more powerful therapeutic successes in cancer immunotherapy (Sau et al., 2018).

In general, functional nanostructures are constructed through a bottom-up approach, in which different molecules clump together into nanosized particles. The forces inducing molecules aggregation are usually covalent bonds from chemical reactions or non-covalent bonds from various weak interactions (Zheng et al., 2018). Non-covalent interactions are weaker than covalent bonds but they widely exist in natural assemblies, like protein structures, DNA double helixes, phospholipid bilayers, and the recognition of ligands with receptors. Supramolecular interactions are a class of interactions categorized by their non-covalent character. Due to its flexibility and reversibility, supramolecular assembly is an appropriate way to construct complicated multi-module hierarchical structures in cancer immunotherapy, similar to building frameworks in LEGO pieces (Huang and Anslyn, 2015; Yang et al., 2015). Moreover, supramolecular-assembled nanostructures make it easier to control multiple modules with the relatively weak and dynamic noncovalent interactions (Ma and Zhao, 2015).

In this mini review, we will summarize some typical supramolecular self-assembled nanostructures in cancer immunotherapy, and describe the modularization idea embodied in the supramolecular assembly. Limited by length, we will mainly introduce some representative nano-assemblies from five typical supramolecular interactions, including supramolecular peptide assembly (SPA), supramolecular DNA assembly (SDA), lipid hydrophobic assembly (LHA), host-guest assembly (HGA), and biomolecular recognition assembly (BRA).

## Supermolecular Assembly for Cancer Immunotherapy

### Supramolecular Peptide Assembly (SPA)

As a subunit of proteins, some peptides can undergo a similar supramolecular assembly by non-covalent intermolecular interactions, including hydrogen bonding, electrostatic interactions, and π-π stacking (Yuan et al., 2019). Moreover, some of the assembled peptides can form similar secondary structure as proteins, like α-helix and β-sheet (Zhang et al., 2020). These natural self-assembly advantages make peptides good assembly modules for the construction of supramolecular nanostructures for cancer immunotherapy (Li et al., 2019; Cai et al., 2020).

With tumor antigens covalently linking to the peptide domains before the assembly or non-covalently incorporated into the peptide together with the assembly, supramolecular peptide assembly has been applied for cancer vaccine construction (Wen and Collier, 2015; Wu et al., 2017). The peptide assembly can not only play a role as a carrier, but may also work as the adjuvant. For example, Collier and co-workers have reported that supramolecular peptide nanofibers assembled from some specific peptide sequences [e.g., QQKFQFQFEQQ (Q11), FKFEFKFE(KFE8)] have self-adjuvanting properties (Rudra et al., 2012; Hudalla et al., 2013). The assembly of peptide domains is essential for the self-adjuvating property, as loss of fibrillization of a peptide module leads to the loss of adjuvant activity. Bacterial lipopeptide Pam_3_-Cys-Ser-Lys_4_ (Pam_3_CSK_4_) is another synthetic peptide adjuvant which is a potent activator of Toll-like receptor 2 and 6. Pam_3_Cys can self-assemble into well-defined nanostructures in aqueous solutions and its coupling with antigen peptide could elicit immune responses without the use of any external adjuvant (Ingale et al., 2007; Cai et al., 2014).

Peptide-assembled nanostructures can also be applied for the loading or conjugating of small molecular immunomodulators for TIME modulation. For example, Cheng et al. designed a sequentially responsive therapeutic peptide for dual-targeted cancer immunotherapy. The peptide contained a short D-peptide antagonist (^D^PPA-1) of PD-L1, and could self-assemble into nanostructures for the loading inhibitor of indoleamine 2,3-dioxygenase (IDO). The nanostructure swelled in a weakly acidic tumor niche due to the protonation of the functional 3-diethylaminopropyl isothiocyanate (DEAP), and released the loaded NLG919 due to the cleavage of the peptide substrate by matrix metalloproteinase-2 (MMP-2) (Cheng et al., 2018). In another study, Han et al. reported a peptide-drug conjugate containing a targeting motif (arginyl-glycyl-aspartic acid, RGD), two protonatable histidines, and an ester bond-linked IDO inhibitor. The conjugate self-assembled into nanostructures in an aqueous solution and exhibited pH-responsive disassembly and esterase-catalyzed drug release after arriving at the tumor tissue, which greatly enhanced the therapeutic potency of PD-L1 blockade in murine Pan02 tumor model (Han et al., 2020).

Peptide materials are biodegradable and normally have good biocompatibility. Their well-defined structure and viability in sequence design also means they have a wide range of functionalities. The assembly behavior not only occurs between peptides, but also appears between peptides and the organism. In an interesting study, Ji et al. utilized a pH low insertion peptide (pHLIP) for anchoring Fc fragments on tumor cell surfaces. The pHLIP can selectively assemble onto the membrane of tumor cells via the conformational transformation in response to the acidic tumor microenvironment, and the inserted Fc fragments or antibodies can subsequently induce antibody-dependent cell-mediated cytotoxicity (ADCC) effects to kill tumor cells (Ji et al., 2019). The recognition between immune cells and the tumor cells is a key step in cancer immunotherapy. This study inspires applying peptide assembly for modifying cell surface and enhancing this recognition effect.

### Supramolecular DNA Assembly (SDA)

Deoxyribonucleic acid (DNA), a type of nucleic acid, is an important biological molecule carrying genetic information, mostly existing as a double helix with another DNA chain. The double helix structure is built through exquisite Watson-Crick base pairing, a non-covalent interaction driven by multiple strong hydrogen bonds. The stable and specific conjugation from two complementary DNA chains is suitable to serve as a linker between two modules, therefore, using supramolecular DNA assembly interactions is another way to construct nanostructures (Chhabra et al., 2010; Chou et al., 2014).

Various DNA nanoassemblies with good stability and high cargo loading capacity have been reported in cancer immunotherapy, and a certain amount of them focus on delivery of CpG—the most commonly used vaccine adjuvant (Chi et al., 2020). These nanostructures include DNA cages, DNA nanotubes, spherical nucleic acids (SNAs), DNA polypods, and so on. For example, DNA tetrahedron architectures assembled from DNA strands can mimic the complex structure of virus-like particles, and provide a multifunctional platform for building DNA vaccines (Liu et al., 2012). SNAs with a solid or hollow core could induce higher inflammatory responses than its linear counterpart due to its special geometry, which generates the ability to target lymph nodes in a high-affinity multivalent fashion (Radovic-Moreno et al., 2015). DNA nano-cocoons (DNCs) were developed through an enzymatic rolling circle amplification method with long-chain single-stranded DNA repeatedly containing interval CpG sequences and cutting sites of restriction enzyme HhaI. In a tumor inflammatory microenvironment, the DNCs are degraded and release the CpG fragments as well as the loaded cargos (anti-PD-1 antibody) to promote synergistic treatment for melanoma (Wang et al., 2016).

The supramolecular interactions between DNA base pairs have also been employed for antibody conjugation on nanostructures to enable modular and tunable control of cell-based cancer immunotherapies. Huang et al. developed an artificial immune cell engager (AICE) nanoplatform for the modular and tunable control of cell-based cancer immunotherapies (Huang et al., 2019). Multiple proteins and antibodies were decorated on the surface of biodegradable nanoparticles via complementary DNA-scaffolding through the direct hybridization. The bases pairing recognition based on multiple hydrogen bonds is very precise and stable, and the hierarchical construction strategy enabled precise and ratiometic loading of multiple cargos on the nanoparticle surface. AICE constructed by this way have been proven to be effective for *ex vivo* expansion of T cells and providing priming signals for systemically administered AND-gate CAR-T cells (Huang et al., 2019).

### Lipid Hydrophobic Assembly (LHA)

Phospholipid and its derivatives are typical amphiphilic molecules that can self-assemble into nanostructures such as liposomes and bilayer sheets through hydrophilic-hydrophobic interactions. Just like the function of vesicles in organisms, liposomes have been widely exploited as nanocarriers to deliver functional molecules (Sercombe et al., 2015). In cancer immunotherapy, liposomes have been widely used for tumor antigens or TIME modulators loading for lymph node or tumor-specific delivery (Kwong et al., 2013; Koshy et al., 2017; Miao et al., 2017; Chen et al., 2019). In addition, the hydrophobic driving forces between lipids can enable “plug in” construction of these cargos. Functional molecules modified with a lipidation motif can be incorporated into the lipid bilayer through lipid hydrophobic interaction in a simple manner. This incorporation strategy presents a new way to construct well-organized multimodular nanostructures.

In a typical example, Moon et al. reported a synthetic high-density lipoprotein nanodiscs composed of phospholipids and apolipoprotein A1-mimetic peptides as a cancer vaccine platform (Kuai et al., 2017). These designed vaccine nanodiscs can easily load antigens and adjuvants by simple incubation with antigen peptides modified with Dioleoyl-sn-glycero-3-phosphoethanolamine-N-[3-(2-pyridyldithio)propionate] (DOPE-PDP) and CpG modified with cholesterol (Cho-CpG), and was shown to elicit up to 47-fold greater frequencies of antigen-specific cytotoxic T-lymphocytes than soluble vaccines (Kuai et al., 2017). Meanwhile, the incorporating types of antigens can be controlled conveniently, making this lipidation incorporation strategy a suitable route for personalized vaccination with patient-specific neoantigens. In another study, they employed the nanodisc to deliver doxorubicin (DOX) for triggering immunogenic cell death (ICD) in the tumor. ICD is a kind of cell death characterized by calreticulin exposure, adenosine triphosphate (ATP), and high mobility group protein B1 (HMGB1) release, which could elicit cell-specific immune responses (Kroemer et al., 2013; Galluzzi et al., 2017). DOX was conjugated to a lipid tail with a pH-sensitive linker, and self-assembled into nanodiscs at mild conditions by simple mixing and incubation. In *in vivo* studies, the delivery of DOX via this way elicited robust antitumor CD8^+^ T cell responses, while the free DOX did not show this effect. The combination of this DOX in nanodiscs plus anti-PD-1 antibody therapy induced complete regression of established murine tumors (Kuai et al., 2018).

Besides small molecule therapeutics, the lipid hydrophobic assembly may also be applied for incorporating auxiliary modules into the liposomes, and enable hierarchical construction of functional structures for cancer immunotherapy. For example, Kulkarni et al. prepared a modular bifunctional therapeutic (anti-SIRPα-AK750) consisting of both signal regulatory protein alpha (SIPRα)-blocking antibodies and colony stimulating factor 1 receptor (CSF-1R) inhibitors by lipid hydrophobic supramolecular assembly to simultaneously block the CD47-SIPRα and MCSF-CSF-1R signaling axis (Kulkarni et al., 2018). Song et al. applied a lipid-protamine-DNA (LPD) nanoparticle for tumor tissue-specific expression of checkpoint inhibition proteins (PD-L1 trap) to reduce the irAEs of anti-PD-L1 antibodies (Song et al., 2018a). The LPD nanoparticle was constructed in a hierarchical self-assembled manner with the inner core firstly formed by the electronic interactions between protamine and DNA, then coated with preformed cationic liposomes, and finally PEG and targeting ligands were modified on the surface by lipid hydrophobic assembly. Surface PEG density can be easily changed to optimize the *in vitro* and *in vivo* behavior of the nanoparticles (Li et al., 1998; Wang et al., 2013). This construction method can be expanded for other systems by changing the DNA plasmid or targeting ligands on the nanoparticle surface (Song et al., 2018b; Wang et al., 2018).

### Host-Guest Assembly (HGA)

The host-guest system first began to gain attention in 1987, along with the first proposal of the concept of supramolecular chemistry (Lehn, 1988). Macrocyclic molecules as the host molecules can bind guest molecules into their cavities via non-covalent forces such as hydrophobic interaction, electrostatic interaction, and hydrogen-bonding interaction, while the external property of the host molecules favors the interaction with surrounding solvents to make the system soluble (Ma and Zhao, 2015). The most commonly used host molecule for supramolecular assembly construction is β-cyclodextrin (β-CD) (Hu et al., 2014; Antoniuk and Amiel, 2016), which has been approved by the US Food and Drug Administration for medical use.

The host molecules can encapsulate hydrophobic drugs as guest molecules into their hydrophobic cavities (Ma and Zhao, 2015). The hydrophobicity of many small molecular immunomodulators limits their direct administration, and sometimes common nanomaterials like liposomes and micelles have only a modest capacity for their incorporation. By modifying macromolecules with β-CD, many hydrophobic small molecular immunomodulators or protein therapeutics can be directly loaded into the cavity of β-CD or entrapped into the nanoassemblies formed by the host-guest interaction between CD and guest molecules like amantadine or azobenzene (Park et al., 2012; Xu et al., 2019; Si et al., 2020). For example, Rodell reported a β-CD nanoparticle (CDNPs) through a reaction between succinyl-β-CD and L-lysine for R848 loading. As a monotherapy, the administered CDNP-R848 could promote the polarization of tumor-associated macrophages into an M1 phenotype (Rodell et al., 2018). Hu et al. prepared a host-guest prodrug nanovectors for combating tumor immune tolerance. Reduction-labile heterodimer of Pheophorbide A (PPa) and NLG919 were integrated with hyaluronic acid via host-guest interactions between β-CD and NLG919. When near infrared laser irradiation was applied, this nanovector could completely eradicate CT26 colorectal tumors through combination immunotherapy (Hu et al., 2020).

### Biomolecular Recognition Assembly (BRA)

The unique interactions between biomolecules for recognition and binding may be used as another supramolecular driving force for nanostructure constructions. This kind of interaction includes antigen-antibody recognition, receptor-ligand recognition, avidin-biotin recognition, and so on (Fritz et al., 2000; Kahn and Plaxco, 2010; Gong et al., 2019). The biomolecular recognition interaction is typically highly specific, with a high affinity and reversibility. For example, the avidin-biotin interaction, which is thought to be the strongest known non-covalent interaction between a protein and a ligand (*K*_d_ = 10^−15^ M), has been applied for the conjugation of biotinylated antibodies to commercial antibiotin-coated microbeads. This design enables microbeads as bispecific engagers with anti-4-1BB and anti-PD-L1 antibodies for blocking the inhibitory checkpoint while simultaneously activating the stimulatory signal (Kosmides et al., 2017), or with MHC-Ig dimer and CD28 antibodies as aAPC to activate and expand tumor-specific T cells (Perica et al., 2014).

The interaction between antigen-antibody recognition has also been developed to modify antibodies on nanostructures. A major interaction applied is through the primary antibody binding to the Fc fragment of the secondary antibody, which will not interfere with the ability of the secondary antibody to bind with antigens. Commercial microbeads coated with anti-mouse IgG1 have been used to conjugate peptide-MHC complexes or clonotypic anti-TCR (1B2) and α-human CD19 as bispecific engagers to redirect T cells to target and destroy tumor cells (Schütz et al., 2014). One of the advantages of this strategy is that the binding site is limited at the Fc domain without interference from the antigen-binding domain and the orientation of the antibody is kept for ligand binding. Inspired by the protein displaying strategies on the inner membranes of bacteria, Kedmi et al. developed a flexible modular platform for non-covalently coating various antibodies on lipid-based nanoparticles. They used a recombinant membrane-anchored lipoprotein (anchored secondary scFv enabling targeting, ASSET) which contains an N-terminal signal sequence for membrane insertion and an scFv for Fc binding. The nanoplatform enables the simple switch of several monoclonal antibodies for diverse leukocytes targeted siRNA delivery (Kedmi et al., 2018).

## Conclusions

**Figure 1:**
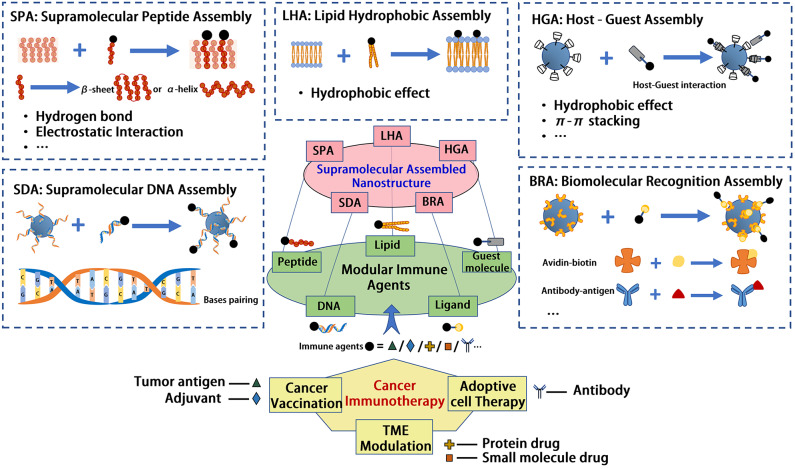
Supramolecular assembled nanostructures for cancer immunotherapy.

Our world is constructed from molecules. While molecular chemistry is concerned with the role of covalent bond in small- and macro- molecules governing the structures, properties, and transformation, supramolecular chemistry is defined as “chemistry beyond the molecule” (Lehn, 1988). Through hierarchical self-assembly, supramolecular chemistry exploits various non-covalent interactions between small- or macro- molecules and manufactures sophisticated organized systems at multiple length scales. Importantly, supramolecular materials and structures are generally tunable, modular, and reversible, as a result of the weak, specific, and multiplicate interactions they use (Webber et al., 2016). These unique properties make supramolecular interactions more attractive in constructing various structures, such as nanostructures, for drug delivery and controlled release. Considering the complexity of nanostructures as well as the therapeutic agents used in immunotherapy, supramolecular assembly provides a biomimetic and cost-efficient way for constructing multimodal nanotherapeutics in immunotherapy, which have been further proved meaningful in expanding the success of cancer immunotherapy. In this mini review, we summarized some of the representative supramolecular nanoassemblies leveraged for cancer immunotherapy, as concluded briefly in Table 1. Five common supramolecular assembled models introduced here are supramolecular peptide assembly (SPA), supramolecular DNA assembly (SDA), lipid hydrophobic assembly (LHA), host-guest assembly (HGA), and biomolecular recognition assembly (BRA), involving supramolecular forces of hydrogen bond, electrostatic interaction, hydrophobic effect, and π-π stacking, etc. The main idea of this construction strategy for nanoimmunotherapeutics is to modularize immune agents with functionalized domains, as shown in Figure 1, to give them the ability to incorporate into the supramolecular nanostructures. Some immune agents serve as the supramolecular assembly modules themselves. For example, some hydrophobic immune modulators can be encapsulated into β-CD as cargo through host-guest assembly, and adjuvant CpG oligodeoxynucleotide can assemble into a DNA backbone directly through bases pairing, while most other immune agents need pre-modifications with supramolecular assembled modules. The summary of these supramolecular assembly strategies may provide some guidance to researchers in using supramolecular interactions in the design of nanostructures for cancer immunotherapy.

**Table 1:** Representative supramolecular assemblies applied in cancer immunotherapy discussed in this paper.

**Supramolecular assembly types**	**Assembly domain**	**Immune agents**	**Immunotherapy types**	**References**
Supramolecular Peptide Assembly (SPA)	Fibrillized Peptide (Q11, KFE8)	Model antigen OVA	Cancer vaccine	Rudra et al., 2012; Hudalla et al., 2013
	Bacterial lipopeptide (Pam_3_CSK_4_)	Peptide antigen	Cancer vaccine	Ingale et al., 2007; Cai et al., 2014
	Amphiphilic peptide (DEAP-^D^PPA-1, NLG-RGD NI)	IDO inhibitor (NLG919), PD-L1 inhibitor	TIME modulation	Cheng et al., 2018; Han et al., 2020
	pH low insertion peptide (pHLIP)	Fc fragment	Cell-based immunotherapy	Ji et al., 2019
Supramolecular DNA Assembly (SDA)	DNA cage	DNA antigen	Cancer vaccination	Liu et al., 2012
	SNAs	Model antigen OVA	Cancer vaccination	Radovic-Moreno et al., 2015
	DNA nano-cocoon (DNCs)	Anti-PD-1 antibody, CpG	TIME modulation	Wang et al., 2016
	DNA base pairs	Anti-CD3, anti-CD28, IL-2, etc.	Cell-based immunotherapy	Huang et al., 2019
Lipid hydrophobic assembly (LHA)	Lipid nanodiscs	Peptide antigens, CpG	Cancer vaccination	Kuai et al., 2017
	Lipid nanodiscs	DOX (inducing ICD)	TIME modulation	Kuai et al., 2018
	Lipid bilayer	anti-SIRPα, CSF-1R inhibitor (BLZ945)	TIME modulation	Kulkarni et al., 2018
	Liposomes	Plasmid DNA	TIME modulation	Song et al., 2018a
Host-guest assembly (HGA)	β-CD	TGF-β receptor-I inhibitor, R848, IDO-1 inhibitor, etc.	TIME modulation	Park et al., 2012; Rodell et al., 2018; Hu et al., 2020
Biomolecular recognition assembly (BRA)	Avidin-biotin	Anti-4-1BB, anti-PD-L1, anti-CD28, MHC-Ig dimer	Cell-based immunotherapy	Perica et al., 2014; Kosmides et al., 2017
	Antigen-antibody, Fc fragment	anti-TCR, anti-CD19	Cell-based immunotherapy	Schütz et al., 2014
	Membrane-anchored lipoprotein (ASSET)	Antibodies against leukocytes, siRNA	Cell-based immunotherapy	Kedmi et al., 2018

With the rapid integration of nanotechnology and cancer immunotherapy, abundant novel nanoimmunotherapeutics have emerged. Due to the weak interaction and assembly from simple structures, supramolecular-based structures offer more potential for clinical translation. For example, an RNA vaccine based on lipid nanoparticles named Lipo-MERIT, an immune agonist CpG with the format of a spherical nucleic acid named AST-008, and nanogels named TRQ15-01 acting as a “backpack” to modify and activate ACT cells have entered phase I clinical trials recently. These three drugs use lipid hydrophobic assembly, supramolecular DNA assembly, and biomolecular recognition assembly, respectively, to construct nanostructures or to combine nanostructures with cells to form larger functionalized structures, suggesting the potential of utilizing supramolecular interactions for cancer immunotherapy. However, compared with traditional nanomaterials, certain differences should be considered in utilizing supramolecular interactions for nanostructure construction as well as clinical translation: (1) as opposed to nanostructures based on amphiphilic copolymers typically prepared by nanoprecipitation or emulsion evaporation method, supramolecular self-assembly is mainly performed in water without the use of organic solvent or surfactants; (2) functional modules for supramolecular assembly are mostly made from simple materials with clear structures, which possess more potential for clinical translation; (3) since supramolecular structures utilize complexity in assembly instead of complexity in molecular structure, the feasibility and controllability of a scaling up of the hierarchical self-assembly system should be a major point for consideration.

## Author Contributions

ZH and WS wrote the manuscript. WS and XC finalized the manuscript.

## Conflict of Interest

The authors declare that the research was conducted in the absence of any commercial or financial relationships that could be construed as a potential conflict of interest.
